# Translating Behavioral Interventions Onto mHealth Platforms: Developing Text Message Interventions for Smoking and Alcohol

**DOI:** 10.2196/mhealth.3779

**Published:** 2015-02-24

**Authors:** Beth C Bock, Rochelle K Rosen, Nancy P Barnett, Herpreet Thind, Kristen Walaska, Robert Foster, Christopher Deutsch, Regina Traficante

**Affiliations:** ^1^Centers for Behavioral and Preventive MedicineAlpert Medical School, Brown UniversityProvidence, RIUnited States; ^2^Center for Alcohol and Addiction StudiesBrown School of Public HealthProvidence, RIUnited States; ^3^Centers for Behavioral and Preventive MedicineThe Miriam HospitalProvidence, RIUnited States; ^4^Live Inspired LLCBethesda, MDUnited States; ^5^Community College of Rhode IslandWarwick, RIUnited States

**Keywords:** mHealth, text message, smoking cessation, alcohol, qualitative methods

## Abstract

The development of mHealth applications is often driven by the investigators and developers with relatively little input from the targeted population. User input is commonly limited to “like/dislike” post- intervention consumer satisfaction ratings or device or application specific user analytics such as usability. However, to produce successful mHealth applications with lasting effects on health behaviors it is crucial to obtain user input from the start of each project and throughout development. The aim of this tutorial is to illustrate how qualitative methods in an iterative process of development have been used in two separate behavior change interventions (targeting smoking and alcohol) delivered through mobile technologies (ie, text messaging). A series of focus groups were conducted to assist in translating a face-to-face smoking cessation intervention onto a text message (short message service, SMS) delivered format. Both focus groups and an advisory panel were used to shape the delivery and content of a text message delivered intervention for alcohol risk reduction. An in vivo method of constructing message content was used to develop text message content that was consistent with the notion of texting as “fingered speech”. Formative research conducted with the target population using a participatory framework led to important changes in our approach to intervention structure, content development, and delivery. Using qualitative methods and an iterative approach that blends consumer-driven and investigator-driven aims can produce paradigm-shifting, novel intervention applications that maximize the likelihood of use by the target audience and their potential impact on health behaviors.

## Introduction

### Background

Mobile Health or “mHealth” is the use of mobile devices to improve health outcomes, health care services, and health related research. The emergence of mobile devices, the swift adoption of these devices across the global population [1], and the rapid expansion of device capability present many challenges for developers of mHealth applications and interventions. One challenge is the complex problem of developing, integrating, and adopting mobile communications into existing health care systems. This includes device management, privacy and security, data quality, workflow integration, interface design, and resistance to change among health care workers [2]. A second set of challenges concern the structure and content of mHealth interventions themselves. Specifically, most mHealth programs have not used behavioral theory to guide intervention development [3-4], although this is changing [5]. In addition, relatively few follow evidence-based principles for health behavior change [6]. This limits our ability to determine *why* an intervention was effective, that is, what the mechanism of action or “active ingredient(s)” might be. A third challenge that has received little attention thus far is that of “technological cultural consistency”, that is ensuring the developed applications and modes of access (including research methodologies) are compatible (culturally consistent) with the ways in which the intended audience uses technology.

To successfully deliver interventions that impact health behaviors and outcomes, mHealth applications should be designed in a manner consistent with the way that individuals use the devices on which the content is to be delivered. Using qualitative methods and an iterative approach that blends consumer-driven and investigator-driven aims can result in the development of paradigm-shifting, novel applications that maximize the likelihood that the intervention will be of use, and will be used by the target audience -thereby maximizing the potential impact on health behaviors and outcomes. However, the development and implementation of mHealth applications has often used a top-down approach driven by the developers and programmers, with relatively little input from the target population [7]. Often, when user-input is requested, it is limited to post-intervention consumer satisfaction ratings or device/application-specific user analytics such as usability testing of the device and its functionality [8-11]. More recently, some investigators have begun to incorporate feedback from the target population during the formative process of developing mHealth interventions [12-13]. However, for mHealth and other technology-based interventions to have a lasting effect on health behaviors, it is not sufficient to develop applications that function as designed and are easy to use (usability) by individuals in the target audience. It is also important, perhaps even crucial, to develop applications that will be stimulating to participants, and what they *want* to use. This process requires input from users from the start of the project and throughout development.

This paper presents examples of mHealth development using qualitative methods in an end-user participatory framework, and demonstrates how use of this model led to a paradigm shift in the approach to behavioral mHealth interventions. This paper describes the results from the formative research of two studies, and the resulting novel approaches to mHealth intervention development.

### Text-2-Quit (T2Q): Changing the Way Tobacco Interventions are Delivered

Each year, smoking kills 443,000 Americans [14]. Currently, 19% of adults in the United States smoke, and adults under age 35 have the highest smoking prevalence of all age groups (25.3%) [14]. Despite the existence of effective, evidence-based smoking cessation therapies and medications, younger adult smokers are least likely to seek treatment, compared to older smokers [15-16]. To see significant reductions in smoking rates, innovative interventions and treatment delivery systems are needed to reach smokers effectively and efficiently.

Use of mobile phones has saturated the general population, and SMS text messaging is widely used, particularly among younger adults (those under age 35) [17-18]. Previous research has shown that even brief behavioral interventions for smoking cessation are effective, and conventional (ie, voice) telephone counseling has long been preferred by a majority of smokers (>75%) compared to face-to-face treatment programs [19], and is often well-received even by unmotivated smokers [20]. Therefore, a logical next step is to adapt smoking interventions for delivery through SMS text messaging. However, there is little theory to support this modality [21], although the evidence base is rapidly expanding.

To date, there have been several studies of text message-based interventions to aid smoking cessation [21-24]. Most of these studies showed significantly greater quit rates among those getting the active text intervention compared with controls. Quit rates among those given text message delivered interventions compare favorably against cessation rates seen for conventional phone (ie, voice) counseling (7-day point prevalence abstinence rate [mean]= 11.1%, rate 8.7-13.4%) [25]. While the majority of interventions using text messaging have demonstrated efficacy, results are not consistent across studies and populations, and there is not yet sufficient data to determine its influence on longer-term smoking abstinence (ie, >6 months) [24, 26]. Importantly, although these programs were largely adapted from evidence-based smoking cessation treatments, little focus has been placed on the characteristics that would be optimal for use within a mobile delivery system.

Text-2-Quit (T2Q) was originally conceived as top-down adaptation of a traditional cognitive-behavioral intervention for smoking cessation that would be delivered through text messages. Core features of interventions for smoking cessation typically include: (1) education about the addictive nature of tobacco and use of medications to aid cessation, (2) identifying “triggers” such as situations and emotions that cue the individual to smoke, (3) setting a definite future “quit day,” (4) problem solving around anticipated difficult situations, (5) enlisting social support, (6) teaching behavioral strategies to break old habits and establish new ones, and (7) one or more face-to-face or telephone (ie, voice) counseling sessions.

The original design for the T2Q intervention included a 2-week program of daily text messages to prepare users for quit day, followed by an 8-week program of text messages beginning with 4 times daily during quit week and tapering to once daily by week 8 post-quit day. The program had different “tracks” to tailor the content, based on the user’s smoking status: “Prepare” (preparing for quit day); “Quit” (quit weeks 1-8); “Not Ready” (designed for those not yet ready for the “Prepare” program); and “Prepare-2” (getting ready for a new quit day after a relapse). Messages in the “Prepare-2” track were similar, but not identical to those in the “Prepare” track, to avoid redundancy and boredom for individuals who had prepared to quit, relapsed, and were now getting ready for a second attempt. The program also allowed users to request additional automated messages to help them deal with immediate cigarette cravings (by texting the keyword “Crave”).

### Formative Research: Asking Targeted Questions of Individuals From the Targeted Population

Formative qualitative research, conducted prior to intervention development or adaptation, enables researchers to understand what representatives of the target audience think about the proposed intervention [27]. It can assess the feasibility of the intervention, identify possible implementation problems and also elicit participant opinions on alternative implementation strategies [28]. The T2Q focus groups were designed to elicit feedback on the planned program content and delivery, so that modifications could be made before the intervention trial. Because little mHealth smoking cessation research had been conducted, we needed to know how people in the target population were using their rapidly evolving mobile technology for communication, and whether they would be willing to use it to engage in a smoking cessation program.

Internet advertisements and flyers posted in local commercial venues were used to recruit participants for focus groups. Individuals calling in response to these ads were screened for eligibility (age 18-35, current smoker or ex-smoker quit less than 1 month, daily user of text messaging). Eligible individuals were invited to attend a single focus group to view a demonstration of the proposed system and provide feedback as potential end-users. Participants (N=21, mean age=25.6, age range= 20-33) included 18 current smokers (mean cigarettes/day=12.8) and 3 individuals who had recently quit (< 3 months) who used text messaging. Prior to the start of the 2-hour focus group all participants completed consent procedures and questionnaires that had been approved by the Institutional Review Board (IRB).

For this study three focus groups were conducted. Focus groups began with a discussion of the participants’ use of computer, social media, and other technologies, narrowing to a discussion of their mobile phone use. Then a short graphical and verbal presentation was given describing the overall problem of smoking, and evidence-based therapies for smoking cessation, followed by the planned design for Text-2-Quit program. The focus group was then opened for more discussion of the planned program itself, using *a priori* semi-structured interview guide to promote discussion of the content and functionality of the intervention. Focus groups were audio-recorded, then transcribed and coded. The agreed upon coding values and transcripts were entered into NVivo10 qualitative data analysis software.

Our analysis of these data showed that there was strong support for a text message-based cessation program. Participants’ suggestions drove us to create a more technologically broad-based program, and led to adjustments to the planned program structure. In particular, participants recommended not only social networking functions, but also more user control of the program —preferably through an online profile “like a Facebook for smokers”, variability in the timing and delivery of messages, and features that would promote additional interaction with the system. Many participants also stated that the program should be able to start on the user’s quit day, even if that day happened spontaneously with no preplanning.

In response to this feedback, the intervention was revised in several ways. First, to enhance user control of the program, additional “key words” were developed that users could text to the program phone number to control their “track” within the program. For example, texting “Slip” increased the number of daily texts to the individual by adding four messages focused on coping with slips and avoiding relapse. Texting “Relapse” would prompt a response asking whether the user was ready to set another quit day (and thus, go into the “Prepare-2” track), or not (and as a result be assigned to the “Not Ready” motivation-focused track). Second, some focus group participants had indicated that receiving messages at standard times would be helpful: “*Something in the beginning of the* day...*right in the morning to motivate you*.” However, others said clearly that receiving messages at fixed times would lead them to ignore the message:

If I know that I’m getting a text at, let’s say, 9:00 in the morning and 5:00 in the afternoon every day, after a while I’m just going to be like “I’m not even going to answer that, [because] I already know what that’s about”

These data showed that participants valued the number and frequency of messages, but also that we needed to vary the timing of messages over the course of the program. Therefore, the intervention was programmed to have both a fixed (start of day, end of day) message delivery and variable timed messages.

Finally, and most significantly, many participants wanted to sign up for this program on the day they decided to quit smoking, rather than in advance of quit day. This response contradicts a significant convention used in most behavioral smoking cessation programs, which are designed to prepare individuals for a specific, future quit date typically several days or weeks after program enrollment. Focus group participants indicated instead that they would sign up for a text message-based program only when they decided that “today’s the day [that I’m ready to quit]”. This feedback led to two important changes: The system was programmed to allow users in any stage of the program to text “Quit” if they decided to quit ahead of their targeted quit day, and they then immediately begin receiving texts appropriate to the first days of quitting. We also wrote post-quit day messages to include the information users may have missed if they had not received the Prepare track of the intervention.

An important theme that emerged from the focus groups was participants’ strong interest in exchanging messages with others enrolled in the intervention. They perceived this feature as another opportunity to interact with the program and have access to social support from others who were trying to quit at key times: “*If there was like a group, you could text and say, ‘I really need a friggin’ cigarette right now*.’” They also wanted the program to provide immediate support for cravings. This was consistent with the way they used phones in other situations …*When I try quitting, you know, I’ll call my husband...[and say] ‘I really want to have a cigarette,’ and he’ll tell me ‘It’s not worth it, just, you know, think about what you can get.’ So you definitely need someone to interact back with you.”*

To address this request, a method was needed that would provide individual social support while simultaneously protecting the privacy of program users. Some previous studies have provided users with contact information to another program user (ie, a “quit buddy”) when requests were made to study staff [22]. However, individuals using technology for social support typically use social networking services and platforms (eg, Twitter). Thus, calling the study staff to receive an individual contact number for a quit buddy is not culturally consistent with the way people use technology. Using online user groups in which users can post messages anonymously (using a UserID) as a model, a separate phone line texting protocol was constructed that allowed individuals to text “Help@*” messages that would be sent to a group comprised of nine other users. It was decided to have 10 members for each @*group to maximize the probability that one or more other users would respond while limiting the chances of an overwhelming number of responses. Conversations in response to these group help messages functioned much like an online chat. By using this protocol filtered through the central phone line, the actual phone numbers of all participants in the group were protected while providing smoking peer contact.

### Process Evaluation: The T2Q Pilot

The Text to Quit (T2Q) study was a randomized controlled trial in which the newly developed smoking cessation texting intervention was tested against a comparison condition in which participants received daily motivational (not smoking related) texts [29]. All procedures for the trial were approved by the Institutional Review Board prior to initiating recruitment. The planned recruitment procedures were similar to those used to recruit the focus groups: advertisements were placed in local media outlets (internet sites, radio programs) asking interested individuals to call or text the study phone. A Research Assistant (RA) contacted callers by voice-phone, provided a brief description of the study (prescreening introduction), and screened callers for study eligibility. Eligible individuals met the following criteria: (1) current daily smoker, (2) interested in quitting smoking in the next 30 days, (3) have a mobile phone with text messaging capability, and (4) use text messaging at least once monthly. Eligible individuals were then scheduled for an in-person baseline visit during which they provided written consent and took part in a single in-person smoking cessation counseling session. Following initial counseling, participants were to be randomized to either the T2Q intervention or the control condition.

Over a period of 3 months 147 contacts (96 calls, 51 texts) were received. However, 28 of those had “text only” phone plans and could not be screened. Eighty-three individuals were contacted by voice-phone for screening, the vast majority were eligible (88%), but most were no longer interested when they were told they would be required to attend an in-person orientation and counseling session. Altogether, a total of 7 participants were enrolled and randomized using these procedures. These slow recruitment and high attrition rates necessitated a change in recruitment methods. In particular, study procedures were needed that were consistent with the way the target audience used technology. Traditional methods of in-person orientation, screening and smoking treatment were clearly not acceptable to most respondents.

Study recruitment was suspended for three months to develop a web portal that would deliver all recruitment procedures seamlessly. The initial web page provided the pre-screening introduction to the study. Interested individuals clicked through to a second page that presented an online screener that was programmed to determine the individual’s eligibility. Eligible individuals were then presented with an online consent form to sign electronically. The online consent included a brief quiz to ensure that individuals understood: that this was a research trial, that participation was voluntary and could be stopped at any time, and that data were confidential. After indicating consent, the participant provided identifying and contact information, and completed an online baseline assessment. Given the length of these procedures and the limitations of attention-span for online surveys, participants were allowed up to two days to return and complete the baseline survey. At the conclusion of the assessment, the web program randomly assigned individuals to the two study arms and presented an online Google calendar that participants used to schedule their counseling session. Users also selected whether they wanted to receive their counseling session in person, by voice phone, Skype, or Google Chat. New advertisements were developed that included the website URL as an alternative to calling the study phone line. Using these methods, 96 individuals were screened, 11 were found ineligible, and 51 signed consent and were randomized over the next 21 days. Study results are published elsewhere [29].

### Summary: Lessons Learned From Text-2-Quit

TXT-2-Quit used an iterative mixed-methods approach in which formative qualitative assessment was an integral, preplanned part of the intervention development and product design. Initial investigator-initiated design was followed by focus groups, and the analysis of qualitative data obtained in those groups was used to modify the design. Following this, the program was implemented and both quantitative data (such as recruitment numbers) and qualitative feedback (conversations with callers regarding why they were unable to participate) resulted in further changes to the program design. From this process several major improvements were made to the program, including adding more user control of program delivery, a peer-to-peer social network for support, and other features that enhanced user-interaction with the program. From a clinical perspective, the most striking change from the original design was allowing for the smoking cessation program to begin on the individual’s quit day, based on individual preference. It also became apparent that using traditional recruitment methods with a new technology-based intervention not optimal. The study was greatly enhanced by revising recruitment and intervention delivery methods to match the way in which the target audience normally uses technology and mobile devices.

## Developing an Alcohol Intervention for Community College Students

Excessive alcohol use is the third-leading preventable cause of death in the United States [30], and is a widespread problem among college students [31]. Nearly half of all community college students (CCS) engage in heavy alcohol use [32], which is similar to the high rates seen among students at four-year colleges [31,33]. However, compared to students at four-year/residential colleges [34], there has been relatively little effort to assess and intervene with community college students on hazardous alcohol use, despite these students comprising 40% of all college students nationwide [35]. Epidemiological and observational studies of CCS have reported high levels of alcohol consumption [36], binge drinking [37-38], and drinking more heavily than students at four-year/residential colleges [39-40]. CCS are also at higher risk for negative consequences of heavy drinking including social and health impairment, physical or sexual assault, and unintentional fatal injuries, and are at significantly higher risk for driving under the influence compared to students at residential colleges [31, 39-40]. CCS also tend to come from low-income families, have more diverse ethnic/racial backgrounds than students at residential colleges [35], and have multiple roles and responsibilities (eg, child rearing, single parents, full and part time employment, etc), which speaks to the need for intervention approaches that are tailored to the needs and life-circumstances of this at-risk population. Intervention delivery modalities, particularly mobile health approaches that can be inexpensively provided in an appealing format with wide reach are particularly compelling for reaching this population.

Text message delivered programs have been developed recently for university students [11] but work with community college students is still lacking. The goal of “Text Message Alcohol Program” (TMAP) was to develop an intervention for alcohol-related harm reduction for community college students delivered using text messaging with components that included motivational messages, harm reduction strategies, evocative questions, and social networking support.

### Formative Research

Phase 1 of the TMAP project involved program development and evaluation and again, formative qualitative work was essential because we needed to know what CCS thought about the planned intervention, including whether they would use it and what they thought of sample messages. CCS from Rhode Island & Southern Massachusetts age 18-28 years, were recruited to participate in the focus groups. Recruitment strategies included campus media outlets, flyers, email, radio advertisements, and presentations in classrooms. Students were eligible if they met the following criteria: (1) age 18-28 years (2) current CCS (3) reported at least three heavy drinking episodes in the past two weeks (4) have a mobile phone and use text messaging at least weekly. A heavy drinking episode was defined as four or more standard drinks for females and five or more standard drinks for males on one occasion in the past two weeks (a standard drink is a 12-ounce beer or wine cooler, a 5-ounce glass of wine, or one mixed drink, or 1 shot of liquor). Of the total 40 participants screened, 26 were found to be eligible for the study. The study was approved by the Institutional Review Board and informed consent was obtained from all participants.

Five focus groups were conducted with a total of 26 students (mean age 22.3 years [SD 3.5]). Each focus group lasted about 2 hours. The focus group guide was developed to learn how students used their phones, including if and when phones were turned off, when students ignored texts, and if they had either mobile phones or text-only services. Plans for the text messaging program and sample messages written by the research team were then shown to participants. These sample messages fell into three categories: safer drinking strategies, myths, and facts about alcohol use, and links to related on-line content. Students were asked for specific feedback on the content of the texts and their preferences for message tone (eg, funny, scary, factual), and format (eg, text only, texts + links to other information). Students were also asked what kind of messages they might text a close friend who was out drinking if they wanted to encourage the friend to be safe about his/her drinking.

As in the T2Q study, focus groups were audio-recorded, transcribed and de-identified. A detailed codebook was constructed based on *a priori* research questions and emergent content. Two research team members individually coded each transcript then met to review the coding. The agreed upon coding values and transcripts were entered into NVivo10 qualitative data analysis software.

CCS reported that messages should apply to specific drinking contexts, including “pre-game” and “post-game” messages for before and after a drinking occasion, and for purposeful drinking (ie, times when drinking is done specifically to get high/drunk). They also said messages should be tailored to the different drinking habits of younger versus older drinkers, and to those who are less experienced with alcohol and its effects, compared with more experienced individuals.

Focus group participants felt that texts should deliver a message of caring. For example: “*Drink responsibly, someone at home loves you*” And *"Hey girl, I hope you have fun tonight, but at the same time, be safe. Enjoy your night.”*

Participants in all the groups indicated that they did not want to be told NOT to drink, but that they did not mind being helped or encouraged to make wise choices when they did drink. And they provided specific feedback on sample messages and why they might not work. For example, in response to the sample message *“Still thirsty? Switch to water. You’ll thank yourself tomorrow!”* participants said that they were not drinking alcohol because they were thirsty, so the message sounded like it was written by people who did not know how or why community college students drink, or how they texted.

It became evident that the intervention should include both fact-based texts to inform and motivate safe drinking as well as texts that sounded like they were written by and for community college drinkers. The research team wrote the first category of texts, and received feedback on them during the focus groups. Methods to craft the second category of texts were informed entirely by the focus group members themselves. During the first group, one participant asked for a paper and pen, and on the spot, began to (re)write texts in his own words. After this occurred, all subsequent groups were provided with note cards and pens and invited (but not required) to revise the researcher written text messages, or to write original messages of their own.

Comparing the students’ texts to the messages written by researchers revealed that although texts are typed, their construction and usage more closely resembles casual speech than written language. The sociolinguist John McWhorter suggests that, in fact, texting is a form of “fingered speech”: a language that has its own structure and specific rules [41]. This new linguistic form is developing and evolving, driven by adolescents and emerging adults who are typing speech and then sending it to one another as text. It is not, therefore likely to be effectively “spoken” by older adults and behavioral scientists.

This realization from the qualitative work led to a redesign of the project procedures, and development of a novel in vivo method of text message content development and collection. An advisory panel of 8 individuals from the target population (CC students who drink and use text messaging) was convened to help construct the actual content of the intervention messages. The advisory panel met once weekly for five weeks. During those meetings and on days between panel meetings, panelists actively composed sample text content using their mobile phones and sent these texts to the study phone line for data collection. Texts were written about the topics that had emerged from the previous focus groups (eg, caring, timing, planning to go out, pre-gaming, morning after messages). Panelists rated a list of factual and strategic messages written by researchers, indicating to what degree each might influence them to engage in safe drinking. They were also asked to rewrite messages to sound more natural –as if they were written by peers. A total of 328 messages were generated by the panelists. These student-generated texts were reviewed by five investigators independently. Messages liked by at least 3 reviewers were included in the pilot randomized controlled clinical trial along with factual and strategic messages that were liked by most panelists. The final program content included 14 panelist-generated messages, 12 factual messages re-written by panelist and 10 strategic messages. The message pool for female participants was modified to include 2 messages about alcohol and sexual safety. These messages are currently being used in the randomized controlled trial.

## Conclusions

Using qualitative methods in an end-user participatory framework produced important changes in the delivery and content of two distinct mHealth interventions designed to be delivered through text messaging. Results of the T2Q study showed that younger adult smokers were interested in participating in a smoking cessation program delivered through text messages. However, qualitative feedback from the target audience regarding the perceived optimal features and structure of a technology-based intervention challenged traditional methods of implementing smoking cessation interventions. Similarly, focus group feedback obtained from community college students about alcohol harm reduction messages was compelling and resulted in using the intervention modality itself (texting) to collect message content examples from students in vivo. That is, messages were created by students texting messages to our phone line both during the focus group itself, and while out in the community during the week between meetings. This in vivo methodology may result in particularly effective message content in that messages written using texting may result in intervention content that “sounds” like real text messages, and may convey a feeling of authenticity to the receiver that content written by other methods may not. To our knowledge, the TMAP study is the first to use this in vivo method of developing text message intervention content.

It may not be sufficient to develop mHealth interventions that function as designed and are usable by individuals in the targeted populations [42]. To have an impact on health and health behaviors, interventions must be perceived by individual users as both useful and desirable. These applications must be something that an individual would *want* to use. To accomplish this, mHealth applications may need to include participation from individuals in the targeted population in the initial design process. Additionally, researchers and developers should document their thought process and rationale for decisions made during the initial stages of protocol and content development [43].  Feedback obtained from these individuals, when obtained early in the design and development process can fundamentally change the organization, delivery and content of planned interventions. Likewise, designing mHealth programs that are culturally consistent with the way individuals already use technology is important. Developing interventions and associated applications in ways that are consistent with how people use technology may result in higher perceived utility and desirability of the final application product and ultimately more efficacious interventions. Further testing will reveal whether these changes result in more used, and therefore, more impactful interventions.

## Abbreviations

CCS: community college students
IRB: Institutional Review Board
T2Q: Text-2-Quit, the smoking cessation text message program developed in this study
TMAP: Text Message Alcohol Program

## References

[1] ICT Switzerland, Geneva: ICT Data and Statistics Division, International Telecommunication Union.

[2] Levin D (2012). MHealth: promise and pitfalls. Front Health Serv Manage.

[3] Chomutare T, Fernandez-Luque L, Arsand E, Hartvigsen G (2011). Features of mobile diabetes applications: review of the literature and analysis of current applications compared against evidence-based guidelines. J Med Internet Res.

[4] Cole-Lewis H, Kershaw T (2010). Text messaging as a tool for behavior change in disease prevention and management. Epidemiol Rev.

[5] De Leon E, Fuentes LW, Cohen JE (2014). Characterizing periodic messaging interventions across health behaviors and media: systematic review. J Med Internet Res.

[6] Abroms LC, Padmanabhan N, Thaweethai L, Phillips T (2011). iPhone apps for smoking cessation: a content analysis. Am J Prev Med.

[7] McCurdie T, Taneva S, Casselman M, Yeung M, McDaniel C, Ho W, Cafazzo J (2012). mHealth consumer apps: the case for user-centered design. Biomed Instrum Technol.

[8] Kizakevich PN, Eckhoff R, Weger S, Weeks A, Brown J, Bryant S, Bakalov V, Zhang Y, Lyden J, Spira J (2014). A personal health information toolkit for health intervention research. Stud Health Technol Inform.

[9] Martínez-Pérez B, de la Torre-Díez I, Candelas-Plasencia S, López-Coronado M (2013). Development and evaluation of tools for measuring the quality of experience (QoE) in mHealth applications. J Med Syst.

[10] Vélez O, Okyere PB, Kanter AS, Bakken S (2014). A usability study of a mobile health application for rural Ghanaian midwives. J Midwifery Womens Health.

[11] Bendtsen M, Bendtsen P (2014). Feasibility and user perception of a fully automated push-based multiple-session alcohol intervention for university students: randomized controlled trial. JMIR Mhealth Uhealth.

[12] Waterlander W, Whittaker R, McRobbie H, Dorey E, Ball K, Maddison R, Myers Smith K, Crawford D, Jiang Y, Gu Y, Michie J, Ni Mhurchu C (2014). Development of an Evidence-Based mHealth Weight Management Program Using a Formative Research Process. JMIR Mhealth Uhealth.

[13] Sharifi M, Dryden EM, Horan CM, Price S, Marshall R, Hacker K, Finkelstein JA, Taveras EM (2013). Leveraging text messaging and mobile technology to support pediatric obesity-related behavior change: a qualitative study using parent focus groups and interviews. J Med Internet Res.

[14] Centers for Disease ControlPrevention (CDC) (2012). Current cigarette smoking among adults - United States, 2011. MMWR Morb Mortal Wkly Rep.

[15] Curry SJ, Sporer AK, Pugach O, Campbell RT, Emery S (2007). Use of tobacco cessation treatments among young adult smokers: 2005 National Health Interview Survey. Am J Public Health.

[16] Hughes JR, Cohen B, Callas PW (2009). Treatment seeking for smoking cessation among young adults. J Subst Abuse Treat.

[17] Smith A Washington, D.

[18] (2011). Smith A.

[19] McAfee TA (2007). Quitlines a tool for research and dissemination of evidence-based cessation practices. Am J Prev Med.

[20] Stead LF, Hartmann-Boyce J, Perera R, Lancaster T (2013). Telephone counselling for smoking cessation. Cochrane Database Syst Rev.

[21] Riley WT, Rivera DE, Atienza AA, Nilsen W, Allison SM, Mermelstein R (2011). Health behavior models in the age of mobile interventions: are our theories up to the task?. Transl Behav Med.

[22] Free C, Knight R, Robertson S, Whittaker R, Edwards P, Zhou W, Rodgers A, Cairns J, Kenward MG, Roberts I (2011). Smoking cessation support delivered via mobile phone text messaging (txt2stop): a single-blind, randomised trial. Lancet.

[23] Rodgers A, Corbett T, Bramley D, Riddell T, Wills M, Lin RB, Jones M (2005). Do u smoke after txt? Results of a randomised trial of smoking cessation using mobile phone text messaging. Tob Control.

[24] Whittaker R, McRobbie H, Bullen C, Borland R, Rodgers A, Gu Y (2012). Mobile phone-based interventions for smoking cessation. Cochrane Database Syst Rev.

[25] The 2008 PHS Guideline Update Panel, Liaisons, Staff Clinical practice guideline.

[26] (2013). The Guide to Community Preventive Services.

[27] Ulin PR, Robinson ET, Tolley EE (2005). Qualitative methods in public health: a field guide for applied research.

[28] Green J, Thorogood N (2013). Qualitative Methods for Health Research. 3rd edition.

[29] Bock B, Heron K, Jennings E, Morrow K, Cobb V, Magee J, Fava J, Deutsch C, Foster R (2013). A Text Message Delivered Smoking Cessation Intervention: The Initial Trial of TXT-2-Quit: Randomized Controlled Trial. JMIR Mhealth Uhealth.

[30] Mokdad AH, Marks JS, Stroup DF, Gerberding JL (2004). Actual causes of death in the United States, 2000. JAMA.

[31] Blowers J (2009). Common issues and collaborative solutions: a comparison of student alcohol use behavior at the community college and four-year institutional levels. J Alcohol Drug Educ.

[32] Hingson RW, Zha W, Weitzman ER (2009). Magnitude of and trends in alcohol-related mortality and morbidity among U.S. college students ages 18-24, 1998-2005. J Stud Alcohol Drugs Suppl.

[33] Wechsler H, Lee JE, Kuo M, Seibring M, Nelson TF, Lee H (2002). Trends in college binge drinking during a period of increased prevention efforts. Findings from 4 Harvard School of Public Health College Alcohol Study surveys: 1993-2001. J Am Coll Health.

[34] Seigers DK, Carey KB (2010). Screening and brief interventions for alcohol use in college health centers: a review. J Am Coll Health.

[35] Provasnik S, Planty M Washington, DC.

[36] Hohman MM (1998). A comparison of alcoholic and non-alcoholic students in a community college addictions program. J Alcohol Drug Educ.

[37] Arliss RM (2007). Cigarette smoking, binge drinking, physical activity, and diet in 138 Asian American and Pacific Islander community college students in Brooklyn, New York. J Community Health.

[38] Sheffield FD, Darkes J, Del Boca FK, Goldman MS (2005). Binge drinking and alcohol-related problems among community college students: implications for prevention policy. J Am Coll Health.

[39] VanKim NA, Laska MN, Ehlinger E, Lust K, Story M (2010). Understanding young adult physical activity, alcohol and tobacco use in community colleges and 4-year post-secondary institutions: A cross-sectional analysis of epidemiological surveillance data. BMC Public Health.

[40] Wall AF, BaileyShea C, McIntosh S (2012). Community College Student Alcohol Use: Developing Context-Specific Evidence and Prevention Approaches. Community College Review.

[41] McWhorter J JK!!!.

[42] Dick JJ, Nundy S, Solomon MC, Bishop KN, Chin MH, Peek ME (2011). Feasibility and usability of a text message-based program for diabetes self-management in an urban African-American population. J Diabetes Sci Technol.

[43] Ybarra ML, Holtrop JS, Bağci Bosi AT, Emri S (2012). Design considerations in developing a text messaging program aimed at smoking cessation. J Med Internet Res.

